# Effects of Short-Duration Artificial Ultraviolet B Exposure on 25-Hydroxyvitamin D_3_ Concentrations in Domestic Rabbits (*Oryctolagus cuniculus*)

**DOI:** 10.3390/ani13081307

**Published:** 2023-04-11

**Authors:** Laure E. Molitor, Kelly Rockwell, Amelia Gould, Mark A. Mitchell

**Affiliations:** Department of Veterinary Clinical Medicine, University of Illinois College of Veterinary Medicine, Urbana, IL 61802, USAmmitchell@lsu.edu (M.A.M.)

**Keywords:** *Oryctolagus cuniculus*, vitamin D, 25-hydroxyvitamin D, ultraviolet B radiation

## Abstract

**Simple Summary:**

Vitamin D is an important hormone that can be acquired through diet or exposure to ultraviolet B (UVB) radiation. Few studies have evaluated the effects of UVB radiation on vitamin D concentrations in the domestic rabbit (*Oryctolagus cuniculus*); however, initial findings have found they can increase their serum 25-hydroxyvitamin D_3_ (25-OHD_3_) following 12 h of artificial UVB exposure. Current husbandry recommendations for rabbits do not include specific UVB lighting requirements. Rabbits are a common pet and research model and are frequently housed indoors without access to natural UVB lighting. Rabbits that are chronically vitamin D deficient may develop mineral deficiencies that can lead to poor calcification of the teeth and skull, predisposing these animals to dental abnormalities, bone infections, and other debilitating diseases. While initial results suggest artificial UVB is positive for rabbits, UVB can also be detrimental to the health of vertebrates. The aim of this study was to determine if shorter-duration UVB exposure could also increase 25-OHD_3_ concentrations. Rabbits were provided 6 h of artificial UVB daily for 14 days, and there was a significant increase in 25-OHD_3_ concentrations over time. These findings affirm that rabbits can use short-duration artificial UVB to increase 25-OHD_3_ concentrations.

**Abstract:**

Vitamin D is an important hormone that can be acquired through diet, exposure to ultraviolet B (UVB) radiation, or a combination of these methods. In domestic rabbits (*Oryctolagus cuniculus*), both methods appear viable, but there is limited research evaluating the effects of UVB on this species. Previous studies found that 12 h of artificial UVB radiation significantly increased 25-hydroxyvitamin D_3_ (25-OHD_3_) concentrations over time. While these findings suggest UVB can be beneficial in rabbits, this form of radiation can also be detrimental to vertebrates. The purpose of this study was to determine if shorter-duration UVB could elicit a similar physiological response in rabbits while minimizing potential negative effects. Six rabbits were used for this pilot study. The baseline serum 25-OHD_3_ was measured for each rabbit and following 14 days of 6 h/day exposure to artificial UVB, a second 25-OHD_3_ sample was collected. There was a significant increase (*p* = 0.001) in serum 25-OHD_3_ over time (Baseline: 27.7 ± 8.1 nmol/L; Day 14: 79.8 ± 9 nmol/L). This study affirmed that 6 h of UVB produced 25-OHD_3_ concentrations similar to those found in rabbits exposed to 12 h of UVB. Future studies should continue to determine how the duration of UVB exposure affects 25-OHD_3_ concentrations.

## 1. Introduction

In the wild, rabbits are exposed to all three beneficial components of sunlight, including ultraviolet, visible, and infrared light. However, current husbandry recommendations for captive domestic rabbits (*Oryctolagus cuniculus*) do not include specific lighting requirements beyond a 12–14 h photoperiod [1,2]. While ambient light in households provides visible light for rabbits, and as endotherms, rabbits do not require special exposure to infrared lighting, recent studies on captive rabbits have demonstrated that artificial ultraviolet B (UVB) light can serve an important role in the endogenous synthesis of vitamin D in these animals [3,4]. In both of these studies, the rabbits were exposed to UVB light for 12 h per day, with Emerson et al. [3] following rabbits over a 14-day trial and Watson et al. [4] over a six-month period. The 25-hydroxyvitamin D_3_ (25-OHD_3_) concentrations were significantly higher in the UVB-exposed rabbits following the 14-day exposure [3], and maintained significantly higher concentrations than the non-UVB-exposed controls over 6 months [4]. Both studies found that captive rabbits exposed to 12 h/day artificial UVB will direct energies to synthesize 25-OHD_3_, suggesting that UVB exposure should be considered for captive rabbits.

While UVB light has been found to play an important role in the endogenous synthesis of vitamin D in vertebrates, it is not without risk, as direct UVB exposure can cause photodermatitis, erythema, structural damage to the eye, and cancer [5,6,7,8,9]. A study evaluating sunlight exposure and subsequent risk of squamous cell carcinoma (SCC) found white cats (*Felis catus*) had a significantly higher risk of developing cutaneous SCC than nonwhite cats, with the greatest effect on the ears and nose, or areas with higher exposure and little to no fur [6]. Laboratory mice (*Mus musculus*) that were chronically exposed to UVB radiation at 280–320 nm were found to develop systemic immunosuppression, which led to the development of primary skin cancer [10]. These findings suggest that studies assessing the level of risk for different species are warranted.

The irradiance and duration of exposure to UVB can increase the risk for adverse effects [6,10]. Emerson et al. [3] did not report any adverse effects in domestic rabbits following a 12 h/day 14-day exposure to artificial UVB; however, screening for negative findings was limited to a physical examination. A similar study was performed on chinchillas (*Chinchilla lanigera*) exposed to 12 h/day of artificial UVB irradiation for 16 days with no reported adverse effects [11]. A longer-term study evaluating the same artificial UVB exposure in domestic rabbits screened the animals for adverse side effects using ophthalmic examinations performed by a board-certified veterinary ophthalmologist and full necropsies with histopathology and found no abnormalities suggesting any pathology associated with the 12 h/day exposure for 6 months [4]. While these studies did not demonstrate any negative side effects, their finite follow-up periods suggest more work is needed to further characterize the risk and value of UVB light for captive rabbits.

The purpose of this study was to evaluate if shorter duration daily exposure to UVB at similar low irradiances to those in previous studies would elicit a similar physiological response in the rabbit while minimizing potential negative effects. The specific hypotheses being tested in this study were that rabbits exposed to 6 h of artificial UVB radiation for 14 days would significantly increase their 25-OHD_3_ concentrations over time compared with their baseline concentrations and that the post-exposure 25-OHD_3_ concentrations following 6 h of exposure would not significantly differ from a previous study following 12 h/day exposure under similar conditions for 14 days [3].

## 2. Materials and Methods

This experimental, non-randomized study was performed under the regulations and policies established by the Institutional Animal Care and Use Committee (IACUC 14-265) at the University of Illinois (Champaign-Urbana, IL, USA). Six dwarf mixed-breed juvenile rabbits (10–12 weeks; 3 females, 3 males) with pigmented haircoats (no albinos) from a private source (Sailfin Pet Shop, Champaign, IL, USA) were used for this study. The sample size used for this study was based on the following a priori information: an alpha = 0.05, a power = 0.8, an expected difference in 25-OHD_3_ concentrations of 50 nmol/L, and a standard deviation (SD) of 15 nmol/L between the baseline and 14-day sampling periods. Each rabbit served as its own control in this experimental study and received the treatment of UVB light exposure. No inter-individual controls (non-UVB-exposed rabbits) were used because all of the available animals (only 6 were available) were required to meet the sample size determination requirements noted in our calculations. As there were no inter-individual controls, we used historic baseline and control data for comparisons (see single-sample *t*-test) to our results.

All the animals were weighed and examined to determine whether they were healthy. The rabbits were housed in same-sex pairs in 71 cm × 44 cm × 41.5 cm plastic-bottomed wire cages (Marchioro S.p.A., Isola Vicentina, Italy) on pine bedding (Sunseed Company, Bowling Green, OH, USA). Fresh water was provided ad libitum in a sipper bottle. The rabbits were offered unlimited timothy grass hay (Western Timothy Hay, Oxbow Animal Health, Murdock, NE, USA) and ¼ cup of timothy-based antibiotic-free pellets daily (Oxbow Animal Health, Murdock, NE, USA). The cage substrate, water, and food were replaced daily. General room lighting was provided by a non-UVB-producing fluorescent light for a 12 h photoperiod. The temperature of the room was maintained at 23–27 °C (73–80.6 °F). The rabbits did not have exposure to artificial UVB lighting or natural sunlight prior to the study.

After the initial acclimation period, each rabbit was anesthetized with 5% isoflurane (IsoFlo; Abbott Laboratories, North Chicago, IL, USA), and 1 L/min oxygen was delivered using a facemask. The rabbits were maintained on 1–2% isoflurane with 1L/min oxygen during blood collection. A total of 1 mL of blood (<1% of total body weight) was collected from the cranial vena cava using a 22- to 25-gauge needle fastened to a 3 mL syringe. All blood collection was performed between 1700 h and 1900 h. Once the sample was collected, each animal was recovered on oxygen and all animals recovered uneventfully. Animal restraint, anesthesia, and venipuncture followed the recommended guidelines outlined in Mitchell and Tully [2].

Blood samples were placed into non-anticoagulant microtainers (Becton Dickinson, Franklin Lakes, NJ, USA) and centrifuged at 4000× *g* for 10 min within 90 min of collection. During the 14-day study, UVB radiation was provided by two full spectrum commercial compact bulbs (23-watt compact bulb, Fluker Farms, Port Allen, LA, USA) externally fixed to the top of the cages. UVB radiation was measured by a radiometer (Solarmeter 6.2; Solar Light Co., Inc., Glenside, PA, USA) within the cage at the level of the animals at 9 points in a grid pattern along the perimeter and directly under the lights on days 1, 8, and 14 from 1700 h to 1800 h. At the distance the bulbs were secured, the rabbits received 15–50 microwatts/cm^2^ of UVB radiation in the range 290–310 nm. UVB exposure was divided equally over two time points (approximately 0600 h–0900 h and 1500 h–1800 h) to mimic exposure at dawn and dusk. Rabbits were observed daily for abnormal behavior and signs of photophobia. On day 14, a second blood sample from each rabbit was collected using the same methods described for baseline sampling. All serum samples were transported on wet ice to the authors’ laboratory and stored at −80° C. Once the second set of samples was collected and processed, all samples (baseline and day 14) were submitted together (within 7 days of second samples being collected) on wet ice to Michigan State University (Diagnostic Center for Population and Animal Health, Lansing, MI, USA) to measure 25-OHD_3_ concentrations using a radioimmunoassay [3,4].

The distributions of the data were evaluated using the Shapiro–Wilk test, skewness, kurtosis, and q-q plots. Because the data were normally distributed, the mean, SD, and minimum-maximum (min-max) values are reported. A repeated measures ANOVA was used to determine if the 25-OHD_3_ concentrations were significantly different over time or by sex; the interaction term time x sex was also included. A single-sample *t*-test was also used to determine if the baseline and 14-day 25-OHD_3_ concentrations were significantly different from a previous study using similar husbandry conditions and artificial UVB lights for 12 h/day for 14 days [3]. SPSS 24.0 (IBM Corp., Armonk, NY, USA) was used to analyze the data. A *p* < 0.05 was used to determine statistical significance.

## 3. Results

All six rabbits served as their own control. The rabbits had mean bodyweights of 411 g (range 350 to 455 g) and 619 g (range 495 to 710 g) at the baseline and 14-day sampling periods. Weight gain was expected due to the age of the animals. There was a significant difference (F = 97.1, *p* = 0.001) in the 25-OHD_3_ concentrations over time for all of the rabbits after being exposed to 6 h of UVB radiation (Table 1); however, there was no significance by sex (F = 0.01, *p* = 0.93) or the interaction term (time × sex; F = 2.8, *p* = 0.167). There was no significant difference in the baseline 25-OHD_3_ concentrations in the rabbits from this study (27.7 nmol/L) compared with those exposed to the same type of artificial light at baseline (baseline control, mean: 29.7; baseline case, 38.8) for those reported in Emerson et al. [3].

## 4. Discussion

The results of this study confirm the authors’ first hypothesis that juvenile rabbits exposed to 6 h of artificial UVB light for 14 days would significantly increase their serum 25-OHD_3_ concentrations. Further, these results confirm our second hypothesis that rabbits can synthesize similar 25-OHD_3_ concentrations using the same type of bulb and husbandry but under a shorter duration of exposure (6 h versus 12 h). These results are important because if the duration/exposure of UVB is shortened and similar 25-OHD_3_ concentrations are achieved, it may be possible to reduce some of the potential adverse effects associated with UVB exposure. Ultimately, longitudinal studies will be needed to confirm the potential risk.

Metabolic bone disease is a common nutritional disorder seen in captive species. Nutritional osteodystrophy results from prolonged deficiencies of calcium or vitamin D or from an inappropriate calcium-to-phosphorus ratio in the diet [12,13,14]. Chronic vitamin D deficiency in rabbits can lead to hypocalcemia and hypophosphatemia [14,15]. Harcourt-Brown proposed hypovitaminosis D may contribute to dental disease, a common health concern in pet rabbits [12,13]. Poor calcification of the teeth and skull can predispose pet rabbits to dental disease, including distorted growth of teeth, enamel hypoplasia, and periosteal penetration of bones of the skull by ectopic tooth roots. These conditions may present to the owner as drooling, anorexia, weight loss, poor grooming, malocclusions, and nasal discharge [12]. Progression of dental abnormalities may result in osteomyelitis; nasolacrimal duct infections; and retrobulbar, maxillary, or mandibular abscesses [12]. Experimental rickets has been produced in juvenile rabbits by feeding a diet deficient in calcium or vitamin D [16]. The juvenile rabbits that were exposed to UVB radiation three times per week or fed a diet that included cabbage exposed to UVB radiation had a protective effect from developing rickets, irrespective of a calcium carbonate included in the diet. A study in skeletally mature rabbits evaluated the effects of feeding a diet deficient in vitamin D and found an increase in severe hypophosphatemia and osteomalacia in comparison with the control group supplemented with vitamin D [14].

Previous studies that have diagnosed rabbits with hypovitaminosis D reported undetectable serum 25-OHD_3_ concentrations in these patients. These vitamin-D-deficient rabbits developed hypocalcemia, hypophosphatemia, and hyperparathyroidism [14,15]. In humans, vitamin D deficiency has been reported at serum 25-OHD_3_ concentrations <20 ng/mL (50 nmol/L) [17,18]. In a group of human patients with osteoporosis, 76% had serum 25-OHD_3_ concentrations <12 ng/mL (30 nmol/L) [18]. Ten rabbits (7%) in a Finnish pet rabbit study were also found to be <12 ng/mL and considered below the limit of severe vitamin D deficiency for humans [19]. Based on the human data, the untreated control rabbits in the Emerson et al. [3] and Watson et al. [4] studies and at baseline in the present study would be considered deficient in vitamin D. These results reinforce the importance of developing a reference interval for 25-OHD_3_ in rabbits to better ascertain the health of these animals and assist with interpreting the results of studies such as the one reported here.

Current lighting recommendations for captive rabbits are limited to maintaining a 12 h photoperiod [1,2,20]; however, the results for the current study suggest that a shorter duration of exposure to UVB light can be used to achieve similar 25-OHD_3_ concentrations to a 12 h UVB exposure. Ultimately, it is important to establish a reference range for 25-OHD_3_ concentrations in domestic rabbits to determine the amount of UVB exposure required to achieve a healthy state. Unfortunately, a larger study population is required to establish a reference range, with a minimum of 20–40 subjects but preferentially >120 study subjects, based on the recommendations of the American College of Veterinary Clinical Pathologists [20]. Because this study was done opportunistically, the authors were limited to the animals that were available. In addition, we were unable to secure enough animals to have a control population. To limit the impact of this shortcoming, the rabbits were managed using the same methods outlined in Emerson et al. [3], and the rabbits served as their own control to reduce intra-subject variation. As the baseline 25-OHD_3_ concentrations were not different between the cases and controls for Emerson et al. [3] and the rabbits in the present study (Table 2), and the only differences in 25-OHD_3_ were noted in the rabbits exposed to UVB, the authors believe the sample size limitation did not impact the results. Additionally, the 25-OHD_3_ concentrations reported in Watson et al. [4] following exposure to the same UVB lights and diet for 12 h/day for 6 months were also not significantly different from the post-UVB exposure values in the rabbits from the present study (present study: 79.8 ± 13.6 nmol/L; Watson et al. [4]: 83.1 ± 22.4 nmol/L; t = 0.54, *p* = 0.62), while the values for the controls were different (present study: 79.8 ± 13.6 nmol/L; Watson et al. [4]: 39.3 ± 26.1 nmol/L; t = 6.7, *p* = 0.003). A direct comparison between baseline 25-OHD_3_ concentrations was not made between Watson et al. [4] and the present study because the initial laboratory diet fed to the rabbits in that study had vitamin D concentrations that were 2.4 times higher than the diet used in the present study and in that of Emerson et al. [3]. Ultimately, the findings in the present study suggest that a shorter duration of UVB may provide similar results for captive rabbits and that longitudinal studies further evaluating the amount of UVB required are warranted.

In addition to considering the length of time an animal is exposed to UVB, it is important to consider the amount (µwatts/cm^2^) of UVB being provided. In the present study, rabbits were exposed to 15–50 µwatts/cm^2^ for 6 h/day. These values are similar to those recorded for Emerson et al. [3] (8.3–58.1 µwatts/cm^2^) and Watson et al. [4] (1–70 µwatts/cm^2^). Due to the similar amounts of UVB exposure, the results of the present study reinforce that a shorter length (6 h/day) of UVB exposure should generate similar 25-OHD_3_ concentrations as 12 h of exposure. To reduce the likelihood of adverse effects, reducing the total quantity of UVB is considered prudent. Future studies should evaluate the effects of lower quantities of UVB, in addition to the length of exposure, to further refine the amount of UVB required to achieve the desired effect in rabbits.

The concept of exposing rabbits to artificial UVB to generate vitamin D is fairly recent [3,4,12]. While there remains much we do not understand, this study, as well as others [3,4], reinforce that artificial UVB can be used by rabbits to synthesize 25-OHD_3_. This is important because rabbits can develop hypovitaminosis in captivity [14,15]. Historically, vitamin D has been provided to captive rabbits through their diet; however, this is not without risk. Hypervitaminosis D is typically linked to dietary sources of vitamin D, with rabbits fed diets containing 3250 to 5000 IU/kg of vitamin D developing calcification of their tissues [21,22,23]. To date, there is no record of hypervitaminosis D associated with UVB exposure [4], and research in humans suggests that there are even mechanisms to destroy previtamin D when UVB exposure is prolonged [24]. The results of the Emerson et al. [3] study noted that the 25-OHD_3_ concentrations did not change in their control group when fed a commercial diet containing vitamin D concentrations considered appropriate for rabbits (900 IU/kg) [21,23,25]. This was also the same diet used in the present study and in Watson et al. [4]. The consistent findings between the baseline 25-OHD_3_ concentrations in the present study and the control rabbits in Emerson et al. [3] and Watson et al. [4] suggest that the dietary levels of vitamin D may be inadequate for captive rabbits. These findings reinforce our need to explore the epidemiology of vitamin D in rabbits by further characterizing how UVB exposure may benefit these animals.

There were no significant differences between the sexes in this study. This was not surprising because the rabbits were juveniles (10–12 weeks) at the time of the study, although these animals were expected to become sexually mature shortly after this age [2]. While the 25-OHD_3_ concentrations in these juvenile rabbits were not significantly different from another population of juvenile rabbits housed under the same conditions [3], they were different from a group fed a high vitamin D diet [4]; thus, it is important to recognize that diet can play an important role and may impact life stages. Kubota et al. found that pregnant does at term that were supplemented with 650 nmol vitamin D had a significantly increased plasma 25-OHD_3_ concentration, as did their kits [26]. As noted previously, high vitamin D concentrations in the diet can have adverse effects on rabbits, including increased fetal mortality or dystrophic mineralization. This represents another important reason for evaluating UVB exposure as a method of increasing vitamin D concentrations rather than evaluating diet. While UVB exposure also carries risk, the findings in this present study reinforce that this risk can be reduced by decreasing UVB exposure from 12 to 6 h to obtain similar results.

In addition to the artificial UVB studies noted previously, there have been recent attempts to characterize the value of natural UVB for captive rabbits. A study in Finland evaluated serum 25-OHD_3_ concentrations in 140 Finnish pet rabbits [19]. Rabbits were divided into groups based on their access to natural UVB radiation, hay, and/or commercial diet. Rabbits with regular access to the outdoors (*n* = 46) had a mean serum 25-OHD_3_ of 27.9 ng/mL (69.75 nmol/L), which is similar to the findings for rabbits with access to artificial UVB in the present study, Emerson et al. [3], and Watson et al. [4]. When compared directly with the results of the present study using a single-sample *t*-test (t = 1.6, *p* = 0.17), there is no significant difference between the two populations. The mean serum 25-OHD_3_ concentrations for all the Finnish pet rabbits (*n* = 140) was 26.0 ng/mL (65 nmol/L), with a range of 4.5–67.5 ng/mL (11.25–168.75 nmol/L). Diet was significantly (*p* = 0.001) associated with serum 25-OHD_3_ concentrations in this population, while access to the outdoors with potential exposure to UVB radiation did not reach statistical significance. There were several limitations to this study that could have influenced the results, including differences in breeds, sexes, ages of rabbits, reliance on owner questionnaires to determine groups, length of outdoor access, types of enclosures, and the ratio of sunny versus shaded areas. Additionally, 15 different brands of commercial feed were provided, with vitamin D concentrations ranging from 700–2000 IU/kg. This cross-sectional study illustrates the complexity of factors that can affect 25-OHD_3_ concentrations in pet rabbits and the importance of further investigating the role of UVB on the health of captive rabbits.

Another study in France utilized an online survey distributed to pet rabbit owners to determine the influence of outdoor exposure on dental disease [27]. The authors did not find an association between outdoor access and the presence of dental disease, but there was statistical significance between dental disease and age. While the authors suggested that exposure to natural UVB was insufficient for preventing dental disease in companion rabbits in France, there were several limitations that could have impacted the results, including the possibility that owners were unable to diagnose subclinical dental disease and that owner reporting on outdoor access could be biased. This study, as well as the Finnish study [19], illustrate the challenges of cross-sectional and survey-based studies and reinforce the need for case-control, cohort, and experimental studies to further elucidate the epidemiology of vitamin D in captive rabbits.

## 5. Conclusions

As expected, the results of this study have generated more questions than answers related to the importance of artificial UVB for captive rabbits. While the authors answered their two hypotheses for this study, that artificial UVB can increase 25-OHD_3_ concentrations in captive rabbits after only 6 h/day of artificial UVB for 14 days and that there is no difference in the 25-OHD_3_ concentrations between 6 h/day exposure and 12 h/day exposure (over 14 days and 6 months of exposure), there remains much still to answer regarding what are normal concentrations of 25-OHD_3_ for captive rabbits and how we can reduce the likelihood of adverse effects associated with UVB exposure in these animals. At this time, based on these results and the results of Watson et al. [4], the authors recommend no more than 6 h of UVB exposure per day for pet rabbits. Additionally, pet rabbit owners should be made aware of the possible adverse side effects associated with UVB light and trained to look for adverse effects such as ocular and dermatologic lesions. Future studies should focus on characterizing the epidemiology of vitamin D in captive rabbits by determining reference ranges for this vital hormone in healthy and in vitamin-D-deficient rabbits, as well as further assessing the roles of artificial and natural UVB on vitamin D synthesis in captive domestic rabbits.

## Figures and Tables

**Table 1:** Rabbit 25-OHD_3_ (nmol/L) concentrations in juvenile domestic rabbits at baseline (*n* = 6) and 14 days later (*n* = 6) after 6 h of artificial UVB exposure per day. The samples collected at 14 days were significantly (*p* = 0.002) higher than the baseline.

Time	Sample	Mean	SD	Min-Max
	baseline	27.7	8.1	17–42
6 h	14 days	79.8	13.6	67–102

**Table 2:** Rabbit 25-OHD3 (nmol/L) concentrations in juvenile domestic rabbits (*n* = 6) at baseline and 14 days later after 6 h/day artificial UVB exposure compared with rabbits exposed to the same artificial lights (irradiance) at baseline (controls, *n* = 6; cases, *n* = 6) and 14 days (controls, *n* = 4; cases, *n* = 5) [3] after 12 h/day exposure.

Time	Study	Group	Duration	Mean	SD	Min-Max	t	*p*
Baseline	Present	Control	N/A	27.7	8.1	17–42		
	Emerson et al.	Control	N/A	29.7	14.9	14–44	0.6	0.57
	Emerson et al.	Cases	N/A	38.8	21.4	15–63	2.2	0.1
14 days	Present	Cases	6 h/day	79.8	13.6	67–102		
	Emerson et al.	Controls	12 h/day	31.7 *	9.9	22–45	7.9	0.001
	Emerson et al.	Cases	12 h/day	66.4	14.3	44–81	2.2	0.09

Abbreviations: N/A, not applicable because no ultraviolet B exposure; SD, standard deviation; min, minimum value; max, maximum value; t, t statistic; *p*, probability; *, significant, *p* < 0.05.

## Data Availability

Data is available upon request from the authors.
